# Density and distribution of the flat mite (*Brevipalpus yothersi*) (*Acari*: *Tenuipalpidae*) on four *Hibiscus* varieties: do leaves tell the full story?

**DOI:** 10.1007/s10493-024-00970-z

**Published:** 2024-12-12

**Authors:** Amy Roda, Gösta Nachman, Katrina Scheiner, Daniel Carrillo

**Affiliations:** 1 United States Department of Agriculture APHIS-PPQ-S&T, Miami, FL USA; 2 Department of Biology, Section of Ecology and Evolution, University of Copenhagen, Copenhagen, Denmark; 3 University of Florida Tropical Research and Education Center, Homestead, FL USA

**Keywords:** Citrus leprosis vector, Sampling methods, Binomial sampling, Population estimate

## Abstract

The flat mite, *Brevipalpus yothersi* (Baker, 1949) is widely distributed in the southern United States. This mite is associated with a citrus leprosis virus ((CiLV-C2) *Cilevirus colombiaense* strain hibiscus) infecting ornamental hibiscus (*Hibiscus rosa-sinensis*) in Florida and Hawaii. Because hibiscus is a highly traded ornamental plant, CiLV-C2 has the potential to spread. In laboratory experiments, the abundance and distribution of *B. yothersi* were studied on four varieties of tropical hibiscus. Plants, grown from cuttings, were infested with female mites, and held in environmental chambers (25 °C, 75% RH). After 3 months, the plants were divided into strata representing the top, middle and bottom of the plant. All mites (including eggs) found on the leaves and woody parts (main stems and side branches) were counted, and the surface area of each part calculated. The number and surface area of leaves and woody parts varied between hibiscus varieties; however, the differences between varieties with respect to total number of mites/plant and mites/cm^2^ were not statistically significant. There were twice as many mites on the woody parts than on the leaves. Most mites were found in the bottom stratum of the plants, and they showed an aggregated spatial distribution. The implications of mite distributions for assessing population sizes of *B. yothersi* were analyzed with the purpose of optimizing sampling without damaging the plant. The efficient sampling protocol developed is an important tool for managing the vector and thereby the disease.

**Supplementary Information:**

The online version contains supplementary material available at 10.1007/s10493-024-00970-z.

## Introduction

The flat mite, *Brevipalpus yothersi* (Baker 1949) is a vector of economically important diseases that have emerged in South America such as citrus leprosis, passion fruit green spot, citrus chlorotic ring spot, hibiscus yellow blotch virus, and Clerodendrum chlorotic spot (Kitajima et al. 2010; Dietzgen et al. 2018; de Lillo et al. 2021; Quito-Avila et al. 2021). Of these diseases, citrus leprosis is considered one of the most damaging by causing fruit blemishing, fruit drop, excessive leaf drop, and dieback of shoots that can kill the tree if not controlled (Rodrigues et al. 2003; Bastianel et al. 2010). Recently, a hibiscus strain of citrus leprosis C2 (CiLV-C2) was found infecting tropical hibiscus plants in Hawaii (Melzer et al. 2013) and Florida (Roy et al. 2018), and infecting passion fruit in Hawaii (Roy et al. 2018; Olmedo-Velarde et al. 2021). The occurrence of CiLV-C2 was initially restricted to a few areas in central Florida, but surveys indicate that the virus has now expanded to additional parts of Central Florida as well as South Florida. The virus appears to be associated primarily with *B. yothersi* and hibiscus; however, a similar virus strain was recently found infecting citrus in Colombia (Roy et al. 2024). Hibiscus spp. are economically important ornamentals (United States Department of Agriculture 2019). Since alternative hosts are important in the epidemiology of plant viruses, hibiscus plants may serve as virus reservoirs, which may lead to infection of other cultivated crops such as citrus (Nunes et al. 2012).

Unlike many plant viruses, *B. yorthersi* vectored viruses do not systemically infect the plant (Bastianel et al. 2010). Therefore, an efficient way to manage these viruses is to control the mite vector. Management of leprosis viruses in Brazil has focused on controlling *Brevipalpus* mites with conventional pesticides, which has led to resistance (Moreira et al. 2022). To mitigate problems associated with resistance, acaricides should be applied using rotations and only when the density of mites exceeds a certain threshold (Nyrop and Binns 1992). Currently, there is a lack of knowledge on how *B. yothersi* mites distribute within hibiscus plants, their aggregation patterns, and how the virus might influence this behavior. In addition, there is limited information on the relationship between the density of *B. yothersi* and the associated damage inflicted on hibiscus plants, which is required to determine intervention thresholds. A prerequisite for identifying such density/damage relationships and treatment thresholds is a reliable method for assessing the number of *B. yothersi* on a hibiscus plant or, alternatively, the average number of mites per unit area.

To develop sampling methods to be used for estimating the number or density of *B. yothersi* on hibiscus plants, we conducted a laboratory experiment where four different hibiscus varieties, known to be susceptible to CiLV-C2, were infested with *B. yothersi*. After three months infestation, we recorded the number of mites occurring on the above-ground plant parts. Based on these data, we compared the four varieties with respect to infestation level to examine whether variability exists in the suitability of hibiscus varieties as hosts for *B. yothersi*. We then investigated how accurately we could estimate the total population of mites inhabiting a plant by counting mites on a small sample of leaves and woody plant parts (i.e. stems and branches) and estimated the sampling time associated with a given sampling procedure. Specifically, we aimed to identify relationships between sampling time and the relative precision of the estimated population size based on samples composed of leaves and woody parts versus samples consisting of only leaves.

Finally, counting mites is often very time consuming and difficult in the field. One way to circumvent this obstacle is to apply a sampling method based on scoring the presence or absence of mites in a sampling unit (Gerrard and Chiang 1970; Wilson and Room 1983; Nachman 1984; Binns and Nyrop 1992). However, before such time-saving methods can be implemented in practice, they must be calibrated and validated against reliable data obtained from sampling programs based on mite counts. Therefore, we used the present data set to investigate whether presence/absence sampling (also called binomial sampling) may serve as an alternative to sampling methods based on counting.

## Methods

Plants and Mites: Cuttings (~ 20 cm long) of four varieties of tropical hibiscus (“Snow Queen”, “Sunny Yellow”, “Seminole Pink” and “President Red”) were placed in potting soil and allowed to root and grow in a climate room for 3 months. Four weeks prior to use in the experiments, the plants were sprayed with a 3% solution of JMS Stylet® paraffinic oil (JMS Flower Farms, Inc. Vero Beach, FL, USA) to remove any arthropods potentially infesting them. The plants were also inspected visually for residual insects and mites. Once verified arthropod free, plants were trimmed to 10 cm and placed in screen cages (93 × 47.5 × 47.5 cm BugDorm; MegaView Science Co., Ltd.; Taichung, Taiwan) held in environmental chambers set at optimal conditions (25 °C, 75 ± 5% RH 12 h Light: 12 h Dark) for *B. yothersi* population growth (Castro-Resendiz et al. 2021). Five environmental chambers were used, with 3 chambers holding 2 replicates each and 2 chambers holding 1 replicate each. The plants were fertilized weekly (Miracle Gro® All Purpose Plant Food, Scotts Miracle Gro Inc., Marysville, OH, U.S.A.) and watered as needed.

*Brevipalpus yothersi,* originally collected from hibiscus plants, were reared on bean (*Phaseolus vulgaris* L.) seedlings for approximately 5 generations as described in Groot and Breeuwer (2006). Leaf disks (10 mm) were cut from clean bean leaves using a cork borer and placed adaxial surface down on a piece of water-saturated cotton held in a petri dish. Thirty adult females taken from the colonies were placed on each disk. Subsequently, each hibiscus plant was infested with mites by placing a single disk with 30 mites on a fully expanded leaf in the middle of the canopy.

Sampling: After 3 months the hibiscus plants were removed from the cages and cut at the base of the plant at the soil surface. Each plant was placed into a zipper seal bag and stored at -20 °C until processed. The frozen plants were divided into thirds representing the bottom (stratum *h* = 1), middle (stratum *h* = 2) and top (stratum *h* = 3) of the plant canopy. The leaves from each stratum were separated from the woody parts. We use the term “stems” to denote both main stems and minor side branches. The stems were cut at the branching points to produce pieces of similar shape and manageable size ($\overline{x }$ = 6.91 cm, *s* = 5.45 cm), termed “stem units”. All adults, immature mites, and eggs occurring in each sampling unit (i.e. a leaf or a stem unit) were counted using a dissecting microscope (Leica M80, Leica Microsystems using10x magnification). The surface area (*A*) of a stem unit with radius *r,* measured with a caliper (Marathon Electronic Digital Caliper, Marthon Watch, Switzerland, range 0–200 mm) and length *l* was calculated as *A* = 2π*rl*. The surface area of a leaf was determined by photocopying each leaf and using a digital image processing software (Rasband 2018) to measure the area of the object.

## Statistical analysis

### Software

All statistical analyses were conducted by means of SAS OnDemand for Academics (SAS Institute 2023), while Excel (Microsoft 2016) was used for simulating sampling strategies and for making graphs.

### Differences between varieties with respect to mite infestation

Kruskal–Wallis tests (PROC NPAR1WAY) were used to compare varieties with respect to the number of mites (sum of eggs, immatures, and adults per plant) and mite density (sum of eggs, immatures and adults per cm^2^ surface area) occurring on leaves, stems and the entire plants. A non-parametric test was selected because of the small sample sizes (8 plants per variety) and the non-normality of count data (Siegel and Castellan 1988). The numbers of eggs, immatures and adults were analyzed separately.

### Vertical distribution of mites

Densities of eggs, immatures, adults, and the total mite density (number of individuals/area), were calculated for each of the three vertical strata. A two-way ANOVA (PROC GLM) was used to analyze a model with density (either total mites or each stage separately) as the dependent variable and with *Variety* and *Stratum*, as well as their interaction, as the independent variables. If neither variety nor the interaction between variety and stratum was significant, these terms were omitted from the analysis, enabling us to analyze differences between the three strata with respect to mite infestation using a Kruskal–Wallis test. Pairwise post-hoc comparisons were carried out by means of Dwass, Steel, Critchlow-Fligner multiple comparison procedure (DSCF option) for mites occurring on the leaves, stems, and the entire plant.

PROC CORR was used to calculate Spearman’s (*r*_*s*_) and Pearson’s (*r*) correlation coefficients to express the correlation between the total number of mites found in the three strata within the plants.

### Distribution of mites between leaves and stems

The number or density of mites occupying the leaves and stems within a given stratum are likely to be positively correlated. To test this hypothesis, we calculated *X*_*h*_ and *Y*_*h*_ as the total number of mites occupying the ${M}_{h}$ leaves and the ${N}_{h}$ stem units, respectively, in stratum *h* for each of the 32 plants. Mite density on leaves in stratum *h* of a given plant (denoted ${x}_{h}$) was calculated by dividing ${X}_{h}$ by the total leaf area of the stratum. Likewise, mite density on the stems in stratum *h* (denoted ${y}_{h}$) was found by dividing ${Y}_{h}$ by the total area of the stems in stratum *h*. We used PROC CORR to obtain Pearson’s and Spearman’s correlation coefficients for the correlation between ${X}_{h}$ and ${Y}_{h}$, and between ${x}_{h}$ and ${y}_{h}$. If the hypothesis of a positive correlation was confirmed, we examined to what extent the number of mites on the stems (${Y}_{h}$) can be predicted from the number of mites found on the leaves (${X}_{h}$) within the same stratum. Plots of ${Y}_{h}$ against ${X}_{h}$ using either arithmetic or logarithmic axes revealed a linearity between the two variables after a logarithmic transformation of both, indicating that the relationship between them could be modeled as

1 $$\ln \hat{Y}_{h} = \alpha + \beta {\text{ln}} X_{h},$$

where *α* is the line’s intercept with the Y-axis and *β* its slope.

The full model also included *Variety*, *Stratum* and the square of $\text{ln} {X}_{h}$ as the independent variables as well as all two-way interactions between the predictor variables*.* Non-significant terms were stepwise omitted from the model until all terms were significantly different from 0 (*P* < 0.05). The model was fitted to data by means of PROC GLM. Since the observed values of ${X}_{h}$ and ${Y}_{h}$ could take 0 values, 1 was added to all values of ${X}_{h}$ and ${Y}_{h}$ prior to the logarithmic transformation. The residuals (*ε*) were checked for normality and variance homogeneity by means of Q–Q plots. Plants without mites were omitted from the analysis. If *β* is not significantly different from unity, Eq. 1 simplifies to

1a $$\ln (\hat{Y}_{h} + 1) = \alpha + {\text{ln}}\left( {X_{h} + 1} \right)$$

We also examined whether the density (mites/cm^2^) on the stems in stratum *h* (${y}_{h}$) can be predicted from the density on the leaves (${x}_{h}$) within the same stratum, similar to the relationship between ${X}_{h}$ and ${Y}_{h}$*.*

### Variation among sampling units with respect to mite abundance

When leaves serve as sampling units, the spatial distribution of mites on leaves within a given stratum can be expressed by means of the spatial variance, calculated as

2 $$s_{h}^{2} = \frac{{\mathop \sum \nolimits_{i = 1}^{{M_{h} }} \left( {x_{hi} - \overline{x}_{h} } \right)^{2} }}{{M_{h} - 1}},$$

where ${M}_{h}$ is the number of leaves in the *h*th stratum of a given plant, ${x}_{hi}$ the number of mites found on the *i*th leaf in the stratum (*i* = 1,2,3,…,${M}_{h}$) and ${\overline{x} }_{h}$ the average number of mites per leaf in the stratum (i.e. ${\overline{x} }_{h}=\frac{1}{{M}_{h}}{\sum }_{i=1}^{{M}_{h}}{x}_{hi})$.

Likewise, the variance of mites per stem unit was calculated as

3 $$s_{h}^{2} = \frac{{\mathop \sum \nolimits_{j = 1}^{{N_{h} }} \left( {y_{hj} - \overline{y}_{h} } \right)^{2} }}{{N_{h} - 1}},$$

where ${N}_{h}$ is the number of stem units in the *h*th stratum of a given plant, ${y}_{hj}$ the number of mites found on the *j*th stem unit in the stratum (*j* = 1,2,3,…,${N}_{h}$) and ${\overline{y} }_{h}$ is the average number of mites per stem unit in the stratum (i.e. ${\overline{y} }_{h}=\frac{1}{{N}_{h}}{\sum }_{j=1}^{{N}_{h}}{y}_{jh})$.

Since the spatial variance (*s*^2^) usually tends to increase with population density (*m*), we used Taylor’s power law (Taylor 1961) to model the relationship between the average density and the variance as

4 $$\log s^{2} = a + b{\text{log}} m$$

If both *a* and *b* are close to unity, it indicates that the species under study tends to be randomly distributed (i.e. ${s}^{2}= m$), whereas a slope significantly larger than 1 indicates that the underlying distribution is aggregated.

The average number of mites per leaf (i.e., ${m}_{h}= {\overline{x} }_{h})$ and stem unit (i.e., ${m}_{h}= {\overline{y} }_{h})$ and the corresponding variances were calculated for each plant and stratum using PROC MEANS. This yielded 192 pairwise values of ${m}_{h}$ and ${s}_{h}^{2}$, but only samples for which both ${m}_{h}$ and ${s}_{h}^{2}$ were different from 0 could be used in the analysis. The pairwise values of log ${m}_{h}$ and log ${s}_{h}^{2}$ were fitted by means of Eq. 4, using PROC GLM. The full model included the covariate log ${m}_{h}$ and the class variables *Variety*, *Stratum* and *PlantPart* (leaf/stem) as well as all two-way interactions between the four predictor variables. Non-significant terms were stepwise omitted from the model until all remaining factors were significant (*P* < 0.05).

### Relationship between mite abundance and proportion of occupied sampling units

A prerequisite for presence-absence sampling (binomial sampling), based on categorizing sampling units according to whether individuals are present or not, is the existence of a quantitative relationship between the density of a population and the proportion of occupied sampling units. Several studies (e.g., Nachman 1984; Hepworth and MacFarlane 1992) have demonstrated that the relationship can be adequately described by the Weibull distribution (Weibull 1953), given as

5 $$p = 1 - e^{{ - \alpha \mu^{\beta } }},$$

where *p* is the proportion of occupied sampling units (out of *n* units), while *μ* is the mean number of individuals/sampling unit. *α* and *β* denote two positive parameters. The underlying distribution is random (Poisson) if *α* = *β* = 1 and aggregated if *β* < 1. Provided Eq. 5 is a realistic description of the relationship, it may be used to predict *μ* from the observed fraction of occupied sampling units (denoted $\widehat{p}$) by rewriting the equation to

6 $$\ln \hat{\mu } = \alpha^{\prime} + \beta ^{\prime}{\text{ln}}( - {\text{ln }}(1 - \hat{p})),$$

where ${\beta }{\prime}=\frac{1}{\beta }$ and ${\alpha }{\prime}=-\frac{1}{\beta }\text{ln}\alpha$. To estimate the parameters of Eq. 6, we replaced $\text{ln}\widehat{\mu }$ with $\text{ln}m$, where *m* denotes the average number of mites per sampling unit. Leaves and stems were analyzed separately. We used data from each stratum of the 32 experimental plants to calculate stratum-specific values of *m* and $\widehat{p}$ (denoted ${m}_{h}$ and ${\widehat{p}}_{h}$), but only strata for which ${m}_{h}>0$ and ${0<\widehat{p}}_{h}<1$ could be used in the analysis. A straight line was fitted to the associated values of $\text{ln}{m}_{h}$ and $\text{ln}(-\text{ln}\left(1-{\widehat{p}}_{h}\right))$ by means of PROC GLM. We also included *Variety* and *Stratum,* as well as the two-way interactions between all the explanatory variables, in the model.

### Development of sampling plans for estimating the total population of *B. yothersi*

Because all leaves and stems from each experimental plant were removed, their surface area measured, and all the mites occupying them counted, these data are assumed to represent the true population sizes and plant areas. Therefore, we can compare the estimates obtained from sampling with the exact values based on a complete census, allowing us to assess both the accuracy and the precision of the estimates (Southwood and Henderson 2021).

Provided the analysis of how mites were distributed within plants reveals that strata contributed significantly to explain the number of mites per sampling unit, stratified random sampling would be superior to simple random sampling with respect to precision (Cochran 1977). In addition, unbiased estimates of population densities or population sizes based on stratified random sampling require that the total number of potential sampling units in each stratum is known (Cochran 1977). Since the time invested in the enumeration of all the leaves and/or stems of a sampled plant can be considerable, especially when the plants are large, the time to count leaves and stem units should be taken into consideration when sampling plans are designed.

### Sampling time

Let us assume that a plant consists of *M* leaves and *N* stem units distributed over three strata (i.e. *L* = 3, $M={\sum }_{h=1}^{L}{M}_{h}$ and $N={\sum }_{h=1}^{L}{N}_{h}$). The number of leaves and stem units sampled from the *h*th stratum are denoted ${m}_{h}$ and ${n}_{h}$, respectively, where ${m}_{h}\le {M}_{h}$ and ${n}_{h}\le {N}_{h}$*.* The average number of mites per unit sampled in the *h*th stratum are denoted ${\overline{x} }_{h}$ and ${\overline{y} }_{h}$ for leaves and stems, respectively. The estimated time invested in sampling a plant is calculated as

7 $$\begin{gathered} T = \mathop \sum \limits_{h = 1}^{L} \left( {m_{h} t_{leaf} + n_{h} t_{stem} } \right) + t_{mite} \mathop \sum \limits_{h = 1}^{L} \left( {m_{h} \overline{x} + n_{h} \overline{y}} \right), \hfill \\ + \mathop \sum \limits_{h = 1}^{L} \left( {\left( {M_{h} - m_{h} } \right)\tau_{leaf} + \left( {N_{h} - n_{h} } \right)\tau_{stem} } \right) \hfill \\ \end{gathered}$$

where the first term accounts for the time it takes to collect the sample, the second term for the time it takes to count the mites, and the last term for the costs associated with enumerating all the leaves and stem units not included in the sample. ${t}_{leaf}$ and ${t}_{stem}$ denote the time per sampling unit to collect, manipulate and inspect a leaf or a stem unit, respectively, even if mites are absent, while ${t}_{mite}$ is the time it takes to count a mite occurring in a sampling unit. Finally, ${\tau }_{leaf}$ and ${\tau }_{stem}$ denote the time it takes to enumerate a leaf or a stem unit, respectively, in order to determine ${M}_{h}$ and ${N}_{h}$.

To determine the time to process a hibiscus sample, cuttings (40–50 cm) were collected from an experimental planting located at the United States Department of Agriculture, Agriculture Research Service, Subtropical Horticultural Research Station, Miami, Florida and taken to the laboratory for processing. The mites and found on each leaf and stem was counted using a dissecting microscope (10x). In addition, the time to search and count each leaf and stem was recorded. This sample provided tentative values of ${t}_{leaf}$, ${t}_{stem}$,${\tau }_{leaf}$, ${\tau }_{stem}$ and ${t}_{mite}$ (Table 3).

### Accuracy and precision of a sampling method

Sampling aims at estimating the true value of a population attribute, e.g. the density of mites per sampling unit. Thus, let $\mu$ denote the true value and $\widehat{\mu }$ the estimate of $\mu$ obtained from sampling. An unbiased sampling method satisfies the condition that the expected value of $\widehat{\mu }$ is *μ*, i.e., *E*($\widehat{\mu })=\mu$. If the sampling method is biased, the expected value of $\widehat{\mu }$ will be *E*($\widehat{\mu })=m$, where *m ≠ μ*. Accuracy of a sample estimate is defined as the expected squared difference between $\widehat{\mu }$ and *μ,* i.e., $E{(\widehat{\mu }-\mu )}^{2}$ obtained as (Cochran 1977)

8 $$MSE\left( {\hat{\mu }} \right) = E\left( {\hat{\mu } - \mu } \right)^{2} = E\left( {\hat{\mu } - m} \right)^{2} + \left( {m - \mu } \right)^{2} = V\left( {\hat{\mu }} \right) + B,$$

where *V*($\widehat{\mu })=E{(\widehat{\mu }-m)}^{2}$ represents the precision and $B={(m-\mu )}^{2}$ the bias associated with $\widehat{\mu }$.

Since $MSE\left(\widehat{\mu }\right)$ tends to increase with $\widehat{\mu }$, we define the relative accuracy as

9 $$RMSE\left( {\hat{\mu }} \right) = \frac{{100\sqrt {MSE\left( {\hat{\mu }} \right)} }}{\mu } \%$$

When *μ* is unknown, which it normally is, we can use the coefficient of variation (also called the relative variance) of $\widehat{\mu }$, given as

10 $$RV\left( {\hat{\mu }} \right) = \frac{{100\sqrt {V(\hat{\mu }} )}}{{\hat{\mu }}} \%$$

to express the relative precision of $\widehat{\mu }$ (Hall et al. 1991; Fidelis et al. 2022), which does not take potential bias into consideration.

### Comparison of two sampling methods

Two different sampling methods to estimate the total number of mites occupying a plant were considered. Method 1 yields unbiased population estimates but requires that at least one leaf and one stem unit are sampled from each stratum, i.e. $1\le {m}_{h}\le {M}_{h}$ and $1\le {n}_{h}\le {N}_{h}$. Method 2 is likely to yield biased population estimates but requires only sampling of leaves (i.e. ${n}_{h}$= 0) from which the estimated number of mites on the leaves in the *h*th stratum is found as ${\widehat{X}}_{h}={M}_{h}{\overline{x} }_{h}$. Instead of counting mites on the stems in the same stratum, their numbers are estimated by means of Eq. 19 (Appendix 1) as

$$\hat{Y}_{h} = \hat{\varphi }\left( {\hat{X}_{h} + 1} \right) = \hat{\varphi }\hat{X}_{h} + \hat{Y}_{0},$$

where $\widehat{\varphi }={e}^{\widehat{\alpha }}$. ${\widehat{Y}}_{0}(=\widehat{\varphi }-1)$ is the expected number of mites on the stems if no mites are found on the sampled leaves. Method 2 is faster and easier to use than Method 1 and will cause less plant damage. However, these advantages should be weighted against the loss of accuracy and/or precision associated with sampling only leaves. Equations relating accuracy and precision of an estimated population size to plant size, mite distribution, and allocation of sampling units are provided for both sampling methods in Appendix 1.

### Simulation of sampling for mites on leaves and stems

To compare the two sampling methods described above, we developed an Excel program (Supplementary material S2) that simulates sampling of mites from plants divided into three vertical strata (i.e., *L* = 3). We used the program to conduct a retrospective analysis of the data obtained from the 24 experimental plants that were occupied by mites. For a given choice of sampling method, sample size and allocation of sampling units among strata, the program calculates the estimated sampling time (Eq. 7), *MSE* (Eq. 8), *RMSE* (Eq. 9) and *RV* (Eq. 10) for each plant. The average values of these statistics were used as a measure of sampling performance (the smaller the values, the better). The total number of leaves ($m=\sum_{h=1}^{L}{m}_{h}$) and stem units ($n=\sum_{h=1}^{L}{n}_{h}$) sampled per plant were either equally distributed among strata (i.e. ${m}_{h}=m/L$*;* ${n}_{h}=n/L$ or optimally allocated among strata, defined as the allocation that minimized the average value of *RMSE.* The optimal allocation for a given sample size was found by means of iteration, using the solver tool in Excel. Since *m*_*h*_ or *n*_*h*_ cannot exceed the actual number of potential sampling units of a plant, the program sets ${m}_{h}$ to ${M}_{h}$ if ${m}_{h}>{M}_{h}$ and ${n}_{h}$ to ${N}_{h}$ if ${n}_{h}>{N}_{h}$.

## Results

### Differences among hibiscus varieties

The four hibiscus varieties differed in plant architecture and surface area (Table 1). The variety *Snow Queen* had more side branches and more and smaller leaves compared to the other varieties. Despite 120–149% more leaves on *Snow Queen* plants, the difference in surface area was smaller, ranging from 30–54% more area compared to the other varieties. The surface area of the stems was also highest for *Snow Queen* plants (38–84% more surface area than the other varieties). *President Red, Seminal Pink*, and *Sunny Yellow* plants were similar with respect to numbers and surface areas of leaves and stems.

**Table 1:** The average (± SE) number of leaves and stem units, the average surface areas of leaves and stems, the total number of plants, and the number of plants infested with *B. yothersi* for each of the four hibiscus varieties used in the experiments

Plant property	Hibiscus variety			
	Seminole pink	President red	Snow queen	Sunny yellow
Average number of leaves/plant	43.13 (5.04)	74.13 (6.86)	292.63 (17.56)	53.00 (6.03)
Average number of stem units/plant	8.38 (1.63)	13.88 (1.47)	26.38 (1.29)	12.50 (1.78)
Average leaf area per plant (cm^2^)	1247.3 (138.9)	1611.9 (165.4)	2176.2 (158.0)	1583.1 (171.6)
Average stem area per plant (cm^2^)	164.1 (13.9)	275.7 (11.9)	399.8 (35.5)	193.7 (19.1)
Number of plants	8	8	8	8
Number of plants with mites	6	6	7	5

**Fig. 1:**
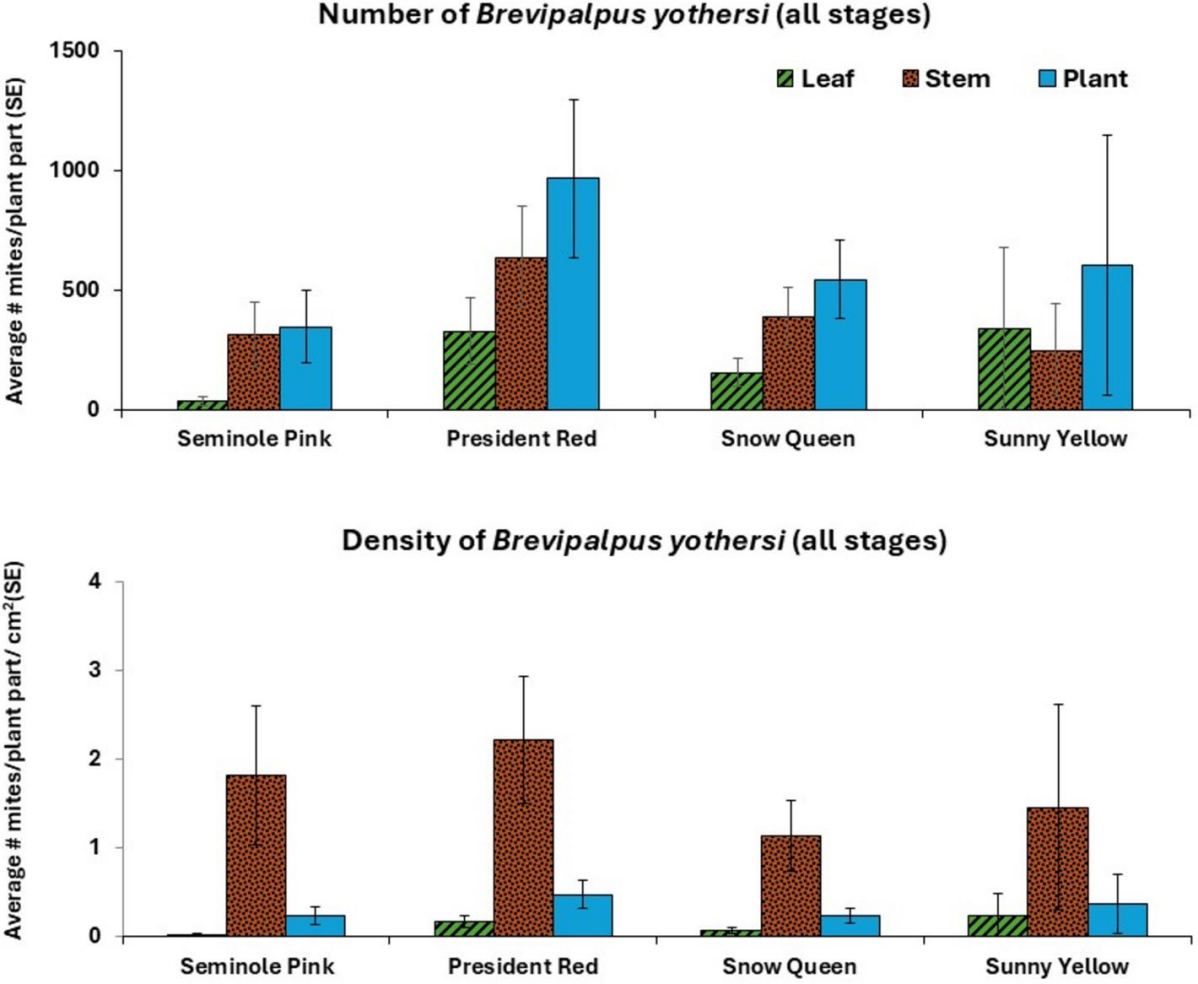
The average number and density of *Brevipalpus yothersi* (eggs, larvae, protonymphs, deutonymphs and adults) found on the leaves, stems, and whole plant of four tropical hibiscus varieties

Mites were found on 24 out of the 32 plants (Table 1). The lack of mites on eight plants is attributed to problems with the infestation procedure, likely because the leaf disk had dropped off the plant before the mites could move away from it. There was no difference between varieties with respect to the number of plants with and without mites (χ^2^ test: ${\chi }_{3}^{2}$ = 1.33; *P* = 0.721). The average numbers of mites (sum of eggs, immatures, and adults) per infested plant (± SE) were 460.7 (± 179.4; *n* = 6), 1289.3 (± 353.2; *n* = 6), 621.9 (± 167.1; *n* = 7) and 941.2 (± 837.1; *n* = 5) on *Seminole Pink*, *President Red*, *Snow Queen* and *Sunny Yellow*, respectively. There was no difference between the four hibiscus varieties with respect to the total number of mites (all stages combined) on leaves (K–W test: ${\chi }_{3}^{2}$ = 5.583; *P* = 0.1338), on stems (K–W test: ${\chi }_{3}^{2}$ = 3.762; *P* = 0.2884), and on entire plants (K-W test: ${\chi }_{3}^{2}$ = 4.066; *P* = 0.2544) (Fig. 1). When the analysis was repeated for the number of each stage separately, there were no differences between the varieties with respect to mite abundance on leaves, stems, and on the entire plant (Supplemental Material S1).

Since the four varieties differed with respect to areas of leaves and stems (Table 1), we also tested whether the varieties differed with respect to densities of mites found on leaves, stems and the entire plants. There was no overall difference between the four varieties with respect to mite densities (all stages combined per cm^2^) on leaves (K–W test: ${\chi }_{3}^{2}$ = 5.545; *P* = 0.1360), on stems (K–W test: ${\chi }_{3}^{2}$ = 2.588; *P* = 0.4596), and on entire plants (K–W test: ${\chi }_{3}^{2}$ = 2.498; *P* = 0.3211) (Fig. 1). When the analysis was repeated for each mite stage separately, there were no differences in their densities on leaves, stems, and the entire plant.

### Vertical distribution of mites

The total number of leaves on the 32 hibiscus plants was 3703, of which 1250 (33.76%) were from the bottom stratum, 1214 (32.78%) from the middle stratum, and 1239 (33.46%) from the top stratum. The total number of stem units was 466, of which 155 (33.26%), 154 (33.05%), and 157 (33.69%) were found in the bottom, middle and top stratum, respectively. Thus, the observed distributions of leaves and stems were close to the presumed distribution, i.e., one third in each stratum. However, the distribution of mites between strata was skewed. Of the 19,559 mites found in total, 10,995 (56.2%) were found in the bottom stratum, 6669 (34.1%) in the middle stratum and 1895 (9.7%) in the top stratum.

**Fig. 2:**
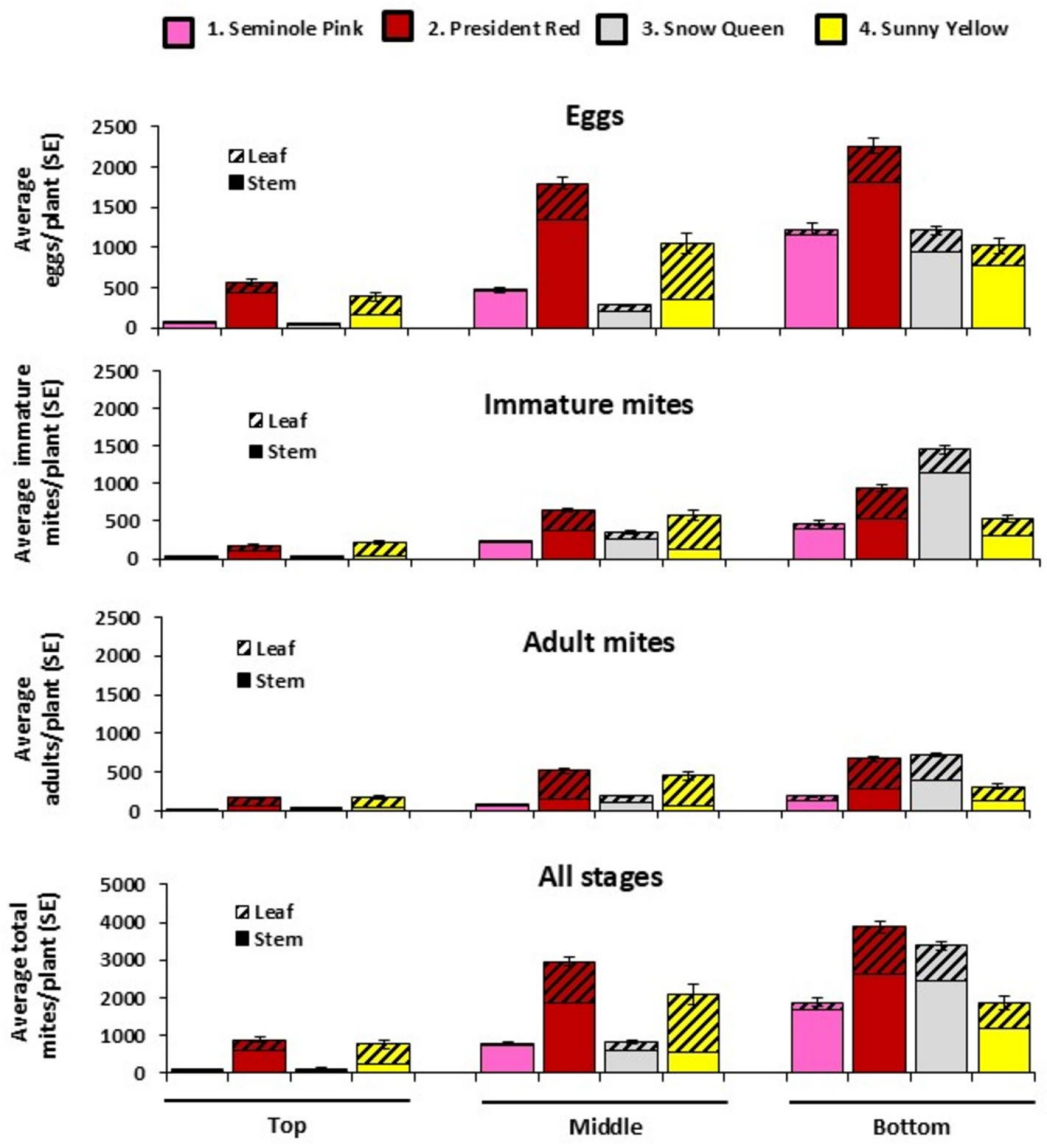
The average number (± SE) of *Brevipalpus yothersi* eggs, immature mites (larvae, protonymph and deutonymph, and adult mites found on the leaves and stems of four varieties of hibiscus

When the density of all mites per stratum was analyzed, the effects of *Variety* and the Variety*Stratum interaction were not significant (GLM: *P* > 0.05) and were therefore omitted from the analysis. The K–W tests revealed significant differences between the three strata with respect to mite density (Mites on leaves: ${\chi }_{2}^{2}$ = 9.672; *P* = 0.0079; Mites on stems: ${\chi }_{2}^{2}$ = 12.86; *P* = 0.0016; Total mites: ${\chi }_{2}^{2}$ = 13.98; *P* = 0.0009). Mite density was highest in the bottom stratum and lowest in the top stratum, with the middle stratum in between (Fig. 2). The densities of mites found in the bottom and top strata were significantly different (Mites on leaves: DSCF = 4.3239; *P* = 0.0063; Mites on stems: DSCF = 4.8458; *P* = 0.0018; Total mites: DSCF = 4.9973; *P* = 0.0012). When each stage was analyzed separately, the results were similar to those obtained when the three stages were combined. Overall, the correlation between the numbers of mites found in two different strata within the same plant was high (Table 2).

**Table 2:** Correlations between strata with respect to numbers of mites (all stages)

Strata	Spearman *r*_*s*_	Pearson *r*
Between middle and bottom Between middle and top Between bottom and top	0.8775 (< 0.0001) 0.8678 (< 0.0001) 0.7806 (< 0.0001)	0.7864 (< 0.0001) 0.9461 (< 0.0001) 0.6807 (0.0003)

Values in parentheses indicate *P-*values based on mites occupying 24 plants

### Distribution of mites between leaves and stems

Of the 19,559 mites found in total on the 24 infested plants, 6,858 (35.1%) were found on the leaves and 12,701 (64.9%) on the stems. 72.6%, 56.2% and 51.6% of the mites inhabiting the bottom, middle and top stratum, respectively, were found on the stems. Adult mites were more evenly distributed between the leaves and stems with 59% of all adult mites being found on leaves. In contrast, only 26% of the eggs and 37% of the immature stages were found on the leaves (Fig. 2).

Based on the 24 infested plants, we found a highly significant positive correlation (*P* < 0.0001) between the 72 pairwise observations of mites on leaves (${X}_{h}$) and stems (${Y}_{h}$) within a stratum (Spearman: *r*_*s*_ = 0.8082; Pearson: *r* = 0.5488). Pearson’s correlation coefficients for each stratum were found as *r*_bottom_ = 0.6291, *r*_middle_ = 0.6207, and *r*_top_ = 0.6872.

**Fig. 3:**
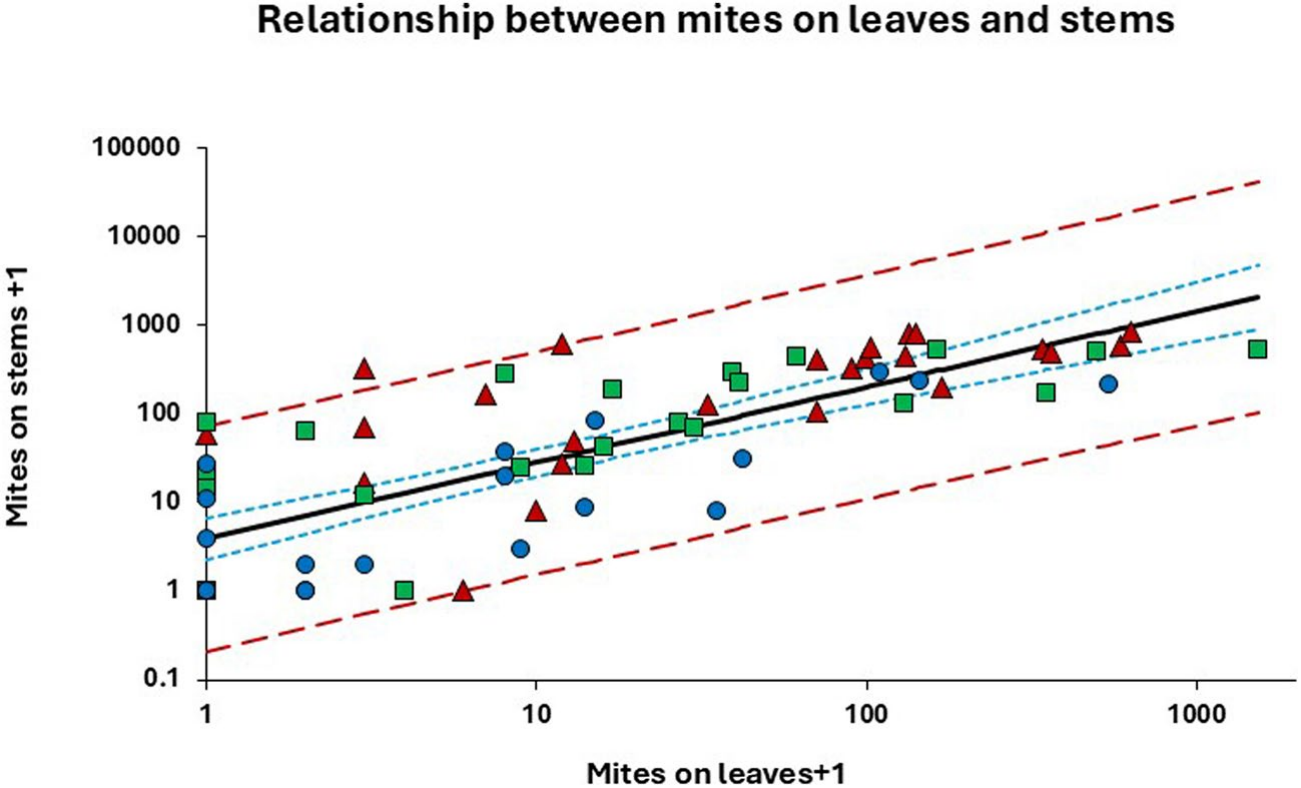
Relationship between the numbers of *Brevipalpus yothersi* (all stages) found on leaves and stems within a given stratum. Symbols indicate observed values sorted by stratum (red triangles = bottom, green squares = middle, blue circles = top). The equation describing the straight line (black line) is given (with SE of estimated parameters in parentheses) as $\text{ln}\left({\widehat{Y}}_{h}+1\right)=\widehat{\alpha }+\widehat{\beta }\text{ln}({X}_{h}+1)$ = 1.3644(0.2703) + 0.8552(0.0802)$\text{ln}({X}_{h}+1)$ (*r*_*adj*_ = 0.7832; *F*_1,70_ = 113.7; *P* < 0.0001). Blue dotted lines: 95% CL for the predicted line; Red dashed lines: 95% CL for individual observations. (Color figure online)

The full model (i.e. Eq. 1 plus all explanatory variables and their interactions) relating the number of mites found on leaves and stems (all stages combined) explained 81.2% of the total variation in the observed values of $\text{ln}({Y}_{h}+1)$ (*F*_23,48_ = 9.01; *P* < 0.0001). However, the only variables contributing significantly to the predict $\text{ln}({Y}_{h}+1)$ were *Stratum* (*F*_2,68_ = 9.73; *P* = 0.0002) and $\text{ln}({X}_{h}+1)$ (*F*_1,68_ = 86.25; *P* < 0.0001). In combination, these two variables explained 80.4% of the total variation (*F*_3,68_ = 53.83; *P* < 0.0001). Though the contribution of *Stratum* was significant, the most important predictor variable was $\text{ln}({X}_{h}+1)$, as this factor alone could explain 61.9% of the variation in $\text{ln}({Y}_{h}+1)$ (*F*_1,70_ = 113.67; *P* < 0.0001). Figure 3 shows the common line fitted to data after omitting all other factors than $\text{ln}({X}_{h}+1)$ from the model. The slope of the line $(\widehat{\beta })$ was found to be 0.8552 (*SE* = 0.0802), indicating that the proportion of mites found on the stems declined with increasing mite densities. Since $\widehat{\beta }$ was not significantly different from unity (${t}_{70}=\frac{\widehat{\beta }-1}{SE(\widehat{\beta })}=\frac{0.8552-1}{0.0802}=-1.805;P=0.0753$), the model was further reduced by setting $\widehat{\beta }$ to 1 (cf. Eq. 1a), yielding

$$\ln \left( {\hat{Y}_{h} + 1} \right) = \hat{\alpha } + \ln \left( {X_{h} + 1} \right) = 0.{9855}\left( {SE = \, 0.{173}0} \right) \, + \ln \left( {X_{h} + 1} \right)$$

which could explain 60.1% of the total variation in the dependent variable.

Since $\widehat{\alpha }$ corresponds to the expected value of $\text{ln}\left({\widehat{Y}}_{h}+1\right)$ when ${X}_{h}=0$, the expected number of mites occupying the stems in a stratum where no mites have been found on the leaves can be estimated from ${\widehat{Y}}_{h}={e}^{\widehat{\alpha }}-1$. Thus, if $\widehat{\alpha }$ = 1.3644 (Fig. 3), the expected value of ${Y}_{h}$ becomes 2.91 (95% CL for the mean: 1.28–5.71; 95% CL for an individual observation of ${X}_{h}=0$: 0–72.43 mites).

The correlation between mite densities (expressed as mites/cm.^2^) on leaves ($${x}_{h}$$) and stems ($${y}_{h}$$) was significant (Spearman: *r*_*s*_ = 0.8011; Pearson: *r* = 0.6827; *n* = 72; *P* < 0.0001). The full model explained 67.9% of the total variation in mite density on the stems ($${y}_{h}$$). When the non-significant interaction terms were excluded, the model explained 61.1%. The influence of *Variety* was marginally significant (*F*_3,65_ = 2.75; *P* = 0.0497) compared with the effect of mite density ($${x}_{h})$$ (*F*_1,65_ = 58.52; *P* < 0.0001)) and *Stratum* (*F*_2,65_ = 7.50; *P* = 0.0012). For a given mite density on leaves ($${x}_{h}$$), the model predicted that the density on the stems ($${y}_{h}$$) will be highest in the bottom stratum and lowest in the top stratum. When a common line was fitted to all data points, its equation was (standard errors in parentheses)

$$\hat{y}_{h} = 1.247\left( {0.288} \right) + 4.629\left( {0.592} \right)x_{h}$$

The straight line explained 46.6% of the total variation in the observed values of ${y}_{h}$ (*F*_1,70_ = 61.09; *P* < 0.0001).

### Variation among leaves and stems with respect to mite abundance

**Fig. 4:**
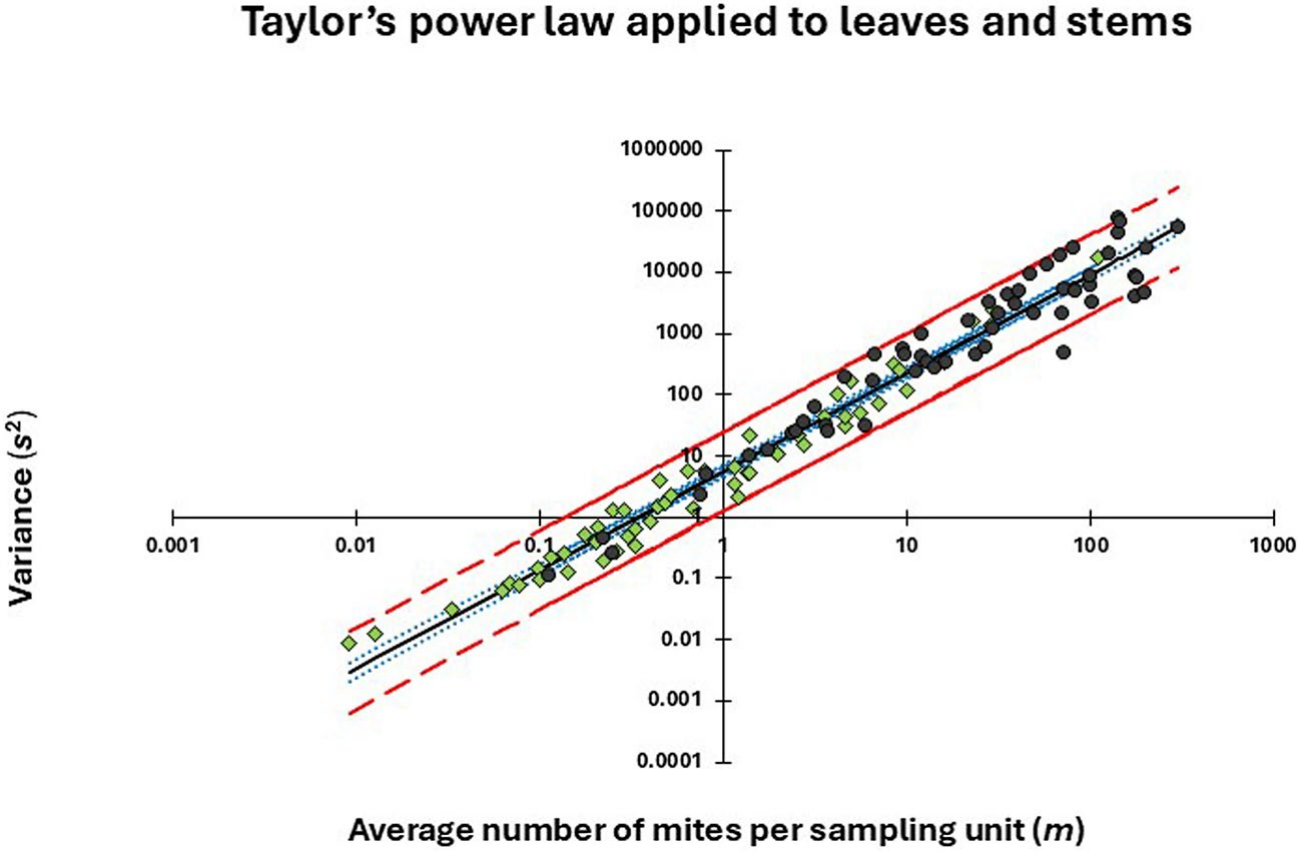
Taylor’ power law fitted to mites on leaves (green diamonds) and stems (brown dots). The black line describes the predicted relationship between $\text{log}{m}_{h}$ and $\text{log}{s}_{h}^{2}$ given as (with standard errors in parentheses) $\text{log}{s}_{h}^{2}=0.7522(0.0350)+1.6070(0.0284)\text{log} {m}_{h}$(*r*_*adj*_ = 0.9833;* F*_1,109_ = 3205.1; *P* < 0.0001). Blue dotted lines: 95% CL for the predicted line; Red dashed lines: 95% CL for individual observations. (Color figure online)

The number of pairwise values of $\text{log}{m}_{h}$ and $\text{log}{s}_{h}^{2}$ available for fitting Taylor’s power was 55 and 56 for leaves and stems, respectively. The full model explained 97.5% of the total variation in the 111 observations of $\text{log}{s}_{h}^{2}$. However, none of the classification variables or their interactions contributed significantly to the model and were therefore omitted. The only remaining factor ($\text{log}{m}_{h}$), explained 96.7% of the total variation in the observed values of $\text{log}{s}_{h}^{2}$. Therefore, a common line was used to describe the relationship between the average number of mites and the variance, irrespective of hibiscus variety, stratum and plant part (Fig. 4). Since the slope (*b*) of the common line was 1.6, and significantly larger than unity, it demonstrates that *B. yothersi* populations were aggregated (Taylor 1961).

### Relationship between mite abundance and proportion of occupied sampling units

To examine how the proportion of leaves or stem units with mites related to the average number of mites per unit, the samples that had at least one mite present were selected for the analysis. For leaves, the sample size available was 53 (out of 96), because mites occupied all leaves in two samples (i.e. ${\widehat{p}}_{h}$ = 1) and were absent in 41 samples (i.e. ${\widehat{p}}_{h}$ = 0). Likewise, mites occupied all stem units in 22 samples and were absent in 38 samples, reducing the sample size from 96 to 36 pairwise values of $m_{h}$ and ${\widehat{p}}_{h}$.

**Fig. 5:**
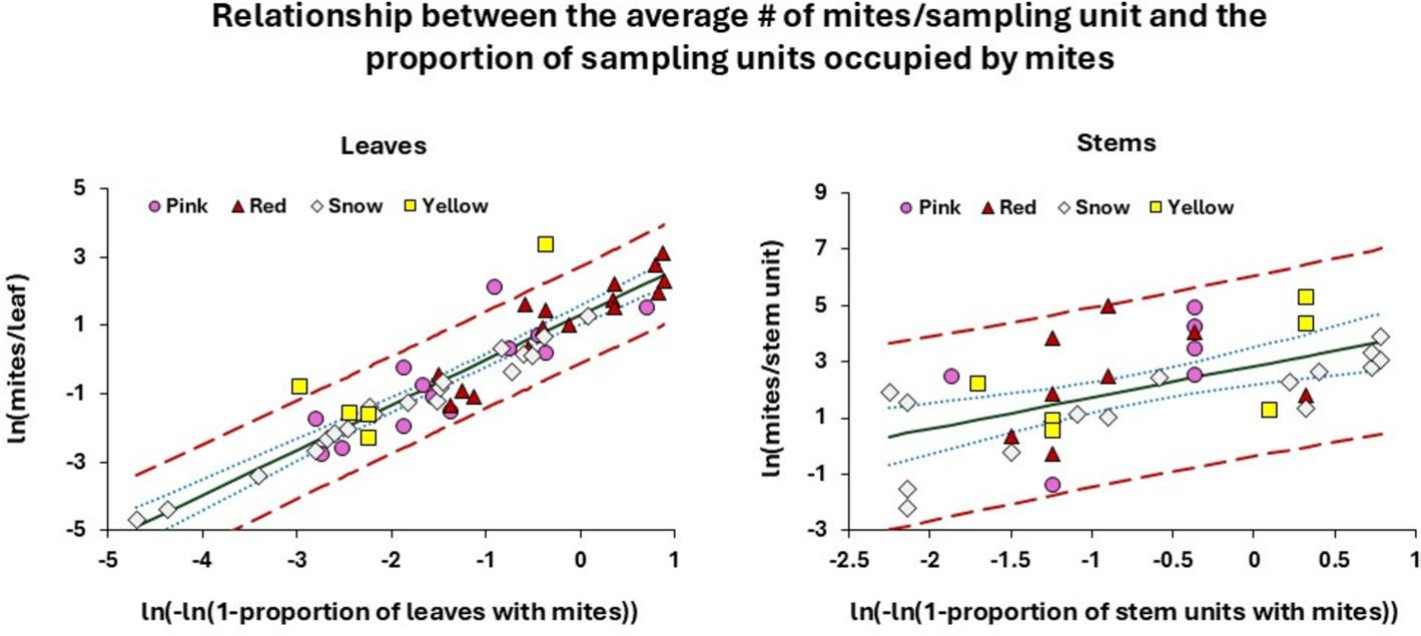
Relationship between the average number of mites/sampling unit (*m*_ℎ_) and the proportion of sampling units occupied by mites (*p̂*_ℎ_) modeled by means of Eq. 6 for leaves and stems. The straight line for the leaves is described by (with SE of estimated parameters in parentheses) *y* = 1.3086(0.1350) + 1.3165(0.0740)*x*, where *y* = ln *m*_ℎ_ and *x* = ln(− ln(1 − *p*_^ℎ_)). The model explained 86.1% of the total variation in the 53 observed values of *y* (*F*_1,51 _= 316.3; *P* < 0.0001). The straight line for the stem units is described by *y* = 2.8471 (0.3239) + 1.1137(0.2800)*x*. It explained 31.8% of the total variation in the 36 observed values of *y* (*F*_1,34_ = 15.82; *P* = 0.0003). Full line: Predicted relationship between ln (− ln(1 − *p*_^ℎ_)) and ln *m*_ℎ_. Blue dotted lines: 95% CL for the predicted line; Red dashed lines: 95% CL for individual observations of ln *m*_ℎ_. (Color figure online)

When the full model was fitted to data for leaves, it explained 91.7% of the total variation in the observed values of $\text{ln}{m}_{h}$ (*F*_16,36_ = 24.96; *P* < 0.0001). Occupation status was by far the most important factor to predict the number of mites/leaf, as this factor alone explained 86.1% of the total variation in $\text{ln}{m}_{h}$. The only other factor that contributed significantly to the model was *Variety* (*F*_3,48_ = 2.95; *P* = 0.0417), but since inclusion of this factor only increased the explained variation with 2.2% (to 88.3%), it justified fitting a common line to all four varieties (Fig. 5). When the same analysis was conducted on mites occupying the stems, the full model predicting the number of mites/unit explained 80.2% of the total variation in $\text{ln}{m}_{h}$ (*F*_16,19_ = 4.81; *P* = 0.0008). Since neither *Stratum* nor *Variety* contributed significantly, a common line was fitted to all data (Fig. 5).

### Development of sampling plans for estimating the total population of *B. yothersi*

**Fig. 6:**
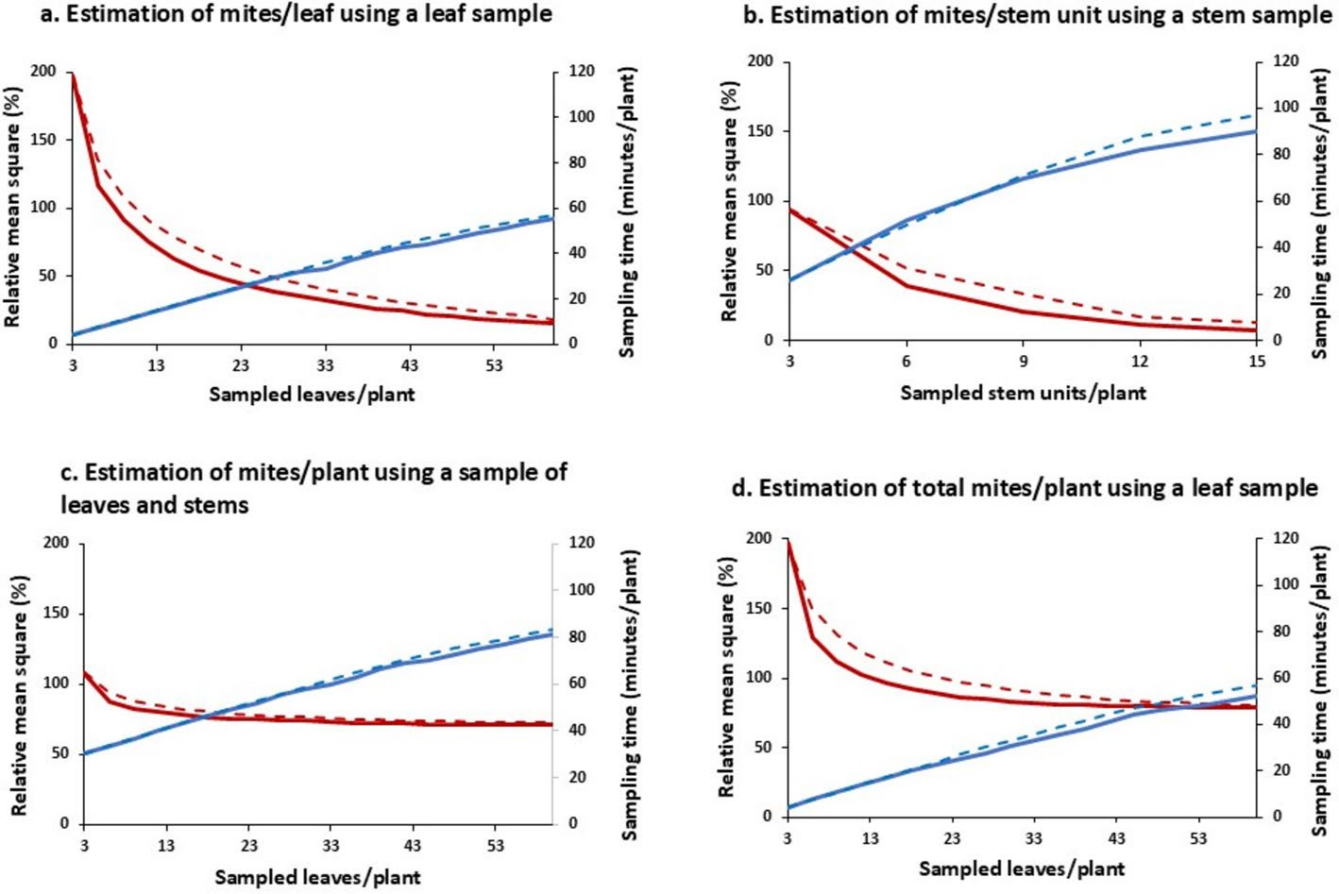
Relative mean square error (red lines) and sampling time (blue lines) when allocation of sampling units was either optimal (solid lines) or equal (dashed lines) among strata. Method 1 was used to estimate mites/leaf (**a**); mites/stem unit (**b**) and mites/plant when the number of stem units/plant (*n*) was fixed to 3 (one per stratum) and *m* leaves were sampled per plant (**c**); only leaves were sampled to estimate mites/plant by Method 2 (**d**). (Color figure online)

The parameter values used to evaluate the performance of a given sampling scenario to assess the abundance of *B. yothersi* on hibiscus plants are presented in Table 3. Figure 6a shows *RMSE* and sampling time (*T*) when the mean number of mites per leaf is estimated from a sample of *m* leaves/plant, while Fig. 6b shows the same statistics for mites/stem in a sample of *n* stem unit/plant. In both cases, *RMSE* declines steadily towards 0 as the sample size increases. A sample based on the optimal allocation of sampling units among strata yields a more accurate population estimate, especially at intermediate sample sizes. Initially, sampling costs (time) increase proportionally with sample size. The rate of increase leveled off as the number of sampling units collected from a stratum approaches the total number of samples available.

**Table 3:** The default parameter values used to evaluate the performance of a given sampling scenario to assess the abundance of *B. yothersi* on hibiscus plants

Parameter definition	Symbol	Estimated value
Scaling parameter of Taylor’s power law^1^ Exponent of Taylor’s power law^2^ Intercept of the calibration line linking mites on stems to mites on leaves^3^ Ratio between numbers of mites on stems and leaves^4^ Variance of the calibration line’s intercept^5^ Time to sample a leaf^6^ Time to sample a stem unit^7^ Time to enumerate a leaf^8^ Time to enumerate a stem unit^9^ Time to count a mite^10^	*a* *b* *α* *φ* *v*(*α*) ${t}_{leaf}$ ${t}_{stem}$ ${\tau }_{leaf}$ ${\tau }_{\text{stem}}$ ${t}_{mite}$	5.652 1.607 0.9855 2.679 0.0299 54 s 385 s 0.53 s 0.89 s 4.75 s

^1^Estimated from the intercept (log *a*) of the line in Fig. 4

^2^Estimated as the slope of the line in Fig. 4

^3^Estimated as the intercept of a line with slope *β* = 1 fitted to data in Fig. 3

^4^Estimated from Eq. 19 as $\widehat{\varphi }= {e}^{\widehat{\alpha }}= {e}^{0.9855}=2.679$

^5^Estimated from Fig. 3 as the square of SE($\widehat{\alpha })$

^6^Estimated from a sample consisting of 50 leaves

^7^Estimated from a sample consisting of 7 stem units

^8^Estimated from enumerating 2806 leaves

^9^Estimated from enumerating 220 stem units

^10^Estimated by counting 180 mites on 50 leaves

For a given sample size, an optimal allocation of sampling units was found to provide an advantage with respect to accuracy and sampling time compared with an equal allocation. Though the actual optimal allocation depends on the sample size, our analysis indicates that an allocation aiming at 60%, 25% and 15% of leaves sampled from the bottom, middle and top stratum, respectively, will be close to the optimal when* m*  > 3. The corresponding values for stems were found to be 70%, 20%, and 10%. In order to obtain an unbiased accurate estimate of the number of mites/plant, it is necessary to sample both leaves and stems. However, we limited the analysis to the special case where only a single stem unit is sampled per stratum (i.e. ${n}_{h}=1$ and *n* = 3) and calculated *RMSE* for increasing numbers of leaves (Fig. 6c)*.* With a single stem unit sampled per stratum, the number of sampled leaves/plant (*m*) had little impact on *RMSE,* as *RMSE* stabilized at around 70%. In comparison, if no stems are sampled at all, Method 2 yields estimates that are slightly less accurate (approximately 80%), but at a much lower cost of time (Fig. 6d).

Although Method 2 yields estimates with a low accuracy (i.e. *RMSE* is high and does not approach 0 even if all leaves are sampled), the relative precision of estimates (*RV*) could be improved by increasing sample size. For example, *RV* decreased from 59.5% when 3 leaves were sampled from each stratum, to 28.9% when 10 leaves were sampled per stratum. When the same number of leaves were sampled per plant, but allocated optimally among strata, the corresponding values were 51.3% (for ${m}_{1}=6$, ${m}_{2}=2$ and ${m}_{3}=1$) and 25.0% (for ${m}_{1}=20$, ${m}_{2}=7$ and ${m}_{3}=3$), respectively.

Total sampling time (*T*) includes the time needed to enumerate all the potential sampling units per plant (${M}_{h}$ and ${N}_{h}$) in order to calculate ${W}_{h}$ and fpc used in the formulae (Appendix 1). In the present case, the experimental plants were small, so the average time to count all the leaves of a plant was approximately 1 min and counting all the stems took on average 10 s/plant. However, if sampling is performed on large mature hibiscus plants, the enumeration of all leaves and stems will be so time-consuming that it should be avoided.

## Discussion

Central to managing vector-transmitted diseases associated with *B. yothersi*, such as the hibiscus strain of citrus leprosis, is the availability of sampling methods that can be used to obtain reliable estimates of the abundance of the vector on its potential host plants. Surveys for *B. yothersi* are difficult because of the mite’s small size, its presence on leaves as well as on other plant parts, and its occurrence in relatively large but scattered populations in the field (Bassanezi and Laranjeira 2007). Efficient sampling programs should aim at maximizing reliability, minimizing sampling effort, and causing little damage to the sampled plants. Our laboratory study of four different hibiscus varieties investigated how these opposite aims can be balanced for sampling *B. yothersi* inhabiting hibiscus plants. Since all mites were counted, their location recorded, and the surface area measured, the data provided unbiased information about how *B. yothersi* was distributed within plants, enabling us to compare different sampling methods with respect to accuracy.

Our study shows that *B. yothersi* could establish and develop on four hibiscus varieties grown under constant environmental conditions. Although the varieties differed in morphology, there was little difference with respect to the level of mite infestation, both in terms of absolute numbers and mites/cm^2^, as well as their distribution within the plants. Most mites were found in the bottom stratum and on the non-foliar plant parts (called stems). Approximately half of all the mites occurring on the plants were found in the lower stratum, about one-third in the middle, and only about 10% in the upper stratum. On these small potted plants made from cuttings, only one-third of the mites occupied the leaves, while the majority inhabited the woody parts. Thus, the ratio between the numbers of mites on the stems and leaves within a stratum (given as $\widehat{\varphi }={e}^{\widehat{\alpha }}$) was estimated to be 2.679. *Brevipalpus yothersi* prefers to find shelter sites to feed (Tassi et al. 2017) and oviposit (Kapp et al. 2024). The cracks, crevices, and other types of irregular surfaces found on stems provide shelter where mites prefer to lay eggs (Andrade et al. 2013), while the adults use leaf veins for shelter when feeding (Tassi et al. 2017). On leaves, damage caused by caterpillars, scale insects and pathogens (i.e. fungi and viruses) are also used by mites for shelter and oviposition (Rodriges et al. 2003). The site selection by *B. yothersi* could be further complicated when acting as a vector for CiLV. Leprosis-transmitting *Brevipalpus* mites are frequently reported on fruits and the presence of CiLV lesions allowed females to oviposit in protective locations, thereby favoring the development of the mites (Kapp et al. 2024). In addition, virus-infected plants may be more attractive and/or more beneficial for vector development (Casteel and Falk 2016; Arena et al. 2016). CiLV infected leaves were found to be more attractive to *B. yothersi* and the mites were less likely to disperse to non-infected leaves (Arena et al. 2016). Thus, the ratio of mites on stems and leaves likely depends on multiple factors, which may change as the plant matures. We expect that *φ* will decline with plant age, reflecting that the structural plant parts (i.e. stems and branches) grow thicker and become more woody, making feeding sites less accessible. Eventually, *φ* might become so small that the mites occurring on the stems can be ignored.

The uneven vertical distribution of *B. yothersi* on the plants justifies the use of stratified random sampling (Cochran 1977). Also, if hibiscus plants are sampled to estimate the total number of *B. yothersi* occupying a plant, mites occurring on both the leaves and the stems should be taken into consideration. Unfortunately, counting mites on stems is cumbersome and requires that the stems and branches be removed and taken to the laboratory in order to count the mites under a binocular microscope. In addition, removal of the stems may inflict considerable damage to the sampled plants. Therefore, an alternative to stem sampling would provide considerable advantages, but may compromise reliability.

We compared two different sampling methods to assess the total number of mites on a plant. Method 1, the protocol to obtain unbiased estimates of population abundance, requires sampling of both leaves and stem units. The alternative method (called Method 2) does not require sampling of stems. We assumed that the number of mites occupying the stems within a given stratum could be predicted from the number of mites found in the foliage within the same stratum. We used a calibration line to assess the expected number of mites on the stems (denoted $\widehat{Y}$) from the number sampled on the leaves (denoted $\widehat{X})$. However, our analysis showed that obtaining an estimate of mites on stems ($\widehat{Y}$) was associated with considerable error, reflected by high values of the relative mean square error (*RMSE*). The error was due to a combination of sampling error associated with assessing the number of mites on leaves ($\widehat{X}$) and the error associated with estimating the individual values of $\widehat{Y}$ by means of a calibration line. Part of the latter error can be attributed to the uncertainty associated with the estimation of the calibration line (Fig. 3). The precision of the calibration line could be improved by increasing the number of samples taken to estimate the line, as this will reduce the variance of the intercept ($v(\widehat{\alpha })$). However, increasing the sample size will not solve the problem if the underlying relationship between $\text{ln}({\widehat{X}}_{h}+1)$ and $\text{ln}({\widehat{Y}}_{h}+1)$ as the relationship is not strictly linear with a slope equal to one, as we assumed in Eq. 1a. Violations of this assumption will bias $\widehat{Y}$ and increase *RMSE.*

Despite the large error associated with estimates of mites per plant based on leaf sampling, the advantage of this method should be balanced against the disadvantage of removing stems. Along with being less damaging to a plant, Method 2 provides a considerable gain with respect to sampling time (Fig. 6c and d). The time saved can be invested in taking larger samples of leaves thereby partially compensating for the low accuracy of Method 2. Programs aiming at surveying populations of flat mites in hibiscus stands should concentrate on leaf sampling, keeping in mind that a part of the total population thereby remains unknown.

The number of sampling units taken per stratum should reflect the variability of mites within the strata to optimize sampling with most units taken in the stratum with the largest variation (Cochran 1977). Based on Taylor’s power law, we have demonstrated that the stratum variances increase exponentially with the average number of mites per sampling unit, indicating that the stratum with the highest mite density will also be the one with the highest variance. Consequently, to optimize sampling, relatively more units should be sampled from the bottom stratum and relatively fewer in the two other strata. Based on the simulations, we found that about 60%, 25% and 15% of the sampled leaves should be taken from the bottom, middle and top stratum, respectively, to optimize sampling. The corresponding values for stem units were 70%, 20% and 10%. However, to identify the optimal allocation of sampling units, it is necessary to know the stratum-specific values of mites per sampling unit (${\widehat{\mu }}_{h}$), the spatial variance (${s}_{h}^{2}$) and the weights (${W}_{h}$) in advance, implying that the optimal allocation can only be identified retrospectively. Without any prior knowledge, the obvious solution is to sample the same number of units in every stratum. However, in studies where the same stand of plants is repeatedly sampled over a long period, retrospective data may help to develop sampling strategies based on an unequal allocation of units, which in the long term may yield more precise estimates than applying an equal allocation.

The total number of units sampled from a plant to assess its infestation of *B. yothersi* depends on the desired level of relative precision (Karandinos 1976; Southwood and Henderson 2021) or the time available for sampling. The lower the value of relative precision (*RV*), the better. Based on Taylor’s power law, *RV* can be found as *RV* $\left(\widehat{\mu }\right)=\sqrt{a{\widehat{\mu }}^{b-2}/n}$ (e.g., Nachman 1984; Southwood and Henderson 2021). Thus, *RV* is expected to decline with increasing sample size (*n*) and/or increasing population density ($\widehat{\mu })$, provided the slope of the relationship *b* < 2. This condition is met by *B. yothersi*, since *b* was estimated to be around 1.6. Our simulations based on data from the 32 experimental plants showed that a precision of around 50% for the mean number of mites per leaf can be achieved by sampling around 20 leaves per plant at a cost of approximately 20 min/plant. However, the sample size must increase to nearly 50 leaves/plant at a cost of about 50 min to attain a precision below 20%.

In the analysis, we have assumed that the total number of leaves (*M*_*h*_) and stem units (*N*_*h*_) in each stratum are known in order to obtain an unbiased estimate of the total number of mites inhabiting a plant. As long as the plants are small, counting all leaves and stem units is manageable, but becomes an overwhelming task when large hibiscus plants are considered. However, if the purpose of sampling is to estimate the mean abundance of mites per leaf or stem unit, we only need to know the stratum weights (${W}_{h}$), defined as the proportion of all sampling units per plant occurring in stratum *h*. The exact stratum weights require information about ${M}_{h}$ and ${N}_{h}$ for all the sampled plants, but in absence of such information we may simply replace ${W}_{h}$ with 1/*L*, where *L* is the number of vertical strata used in the sampling program. In addition, when plants are large, the finite population correction factor (fpc = $1-{n}_{h}/{N}_{h}$) occurring in Eq. 15 can be set to 1.

Because counting mites and other small arthropods is usually very laborious and difficult in the field, methods aiming at reducing sampling time have been suggested, such as sequential sampling (e.g., Kuno 1969; Green 1970), presence-absence (binomial) sampling (e.g., Gerrard and Chiang 1970; Nachman 1984; Schaalje et al. 1991; Hepworth and MacFarlane 1992), or a combination of both methods (e.g. Nyrop et al. 1994; Knapp et al. 2006; Ouassat 2019). Presence-absence sampling offers a way to evade counting in large-scale surveys once a calibration line linking the proportion of occupied sampling units (*p*) to the mean number of individuals per unit (*m*) has been determined. Our data indicate that presence-absence sampling may serve as a shortcut for estimating mites on leaves, but not on the stems. The large scatter associated with stem samples can be attributed to the fact that stem units vary with respect to age, length, diameter, position etc. In contrast, leaves are more uniform when they originate from the same variety of hibiscus, and may, therefore, better serve as standardized sampling units. In addition, since presence-absence sampling requires that sample values of *p* take values between 0 and 1, this condition is more likely met with leaf sampling, because it is possible to take a larger sample of leaves than of stems. Finally, while it is possible to inspect a leaf for presence of mites without detaching it from the plant, this may not be so easy with respect to the woody parts.

## Conclusion

The development of an efficient sampling protocol is an important tool for managing the vector and, ultimately, the viruses associated with the vector. Sampling to obtain accurate estimates of the total number of *B. yothersi* inhabiting full-grown hibiscus plants is unrealistic. Instead, it is necessary to focus on the mites inhabiting the leaves and accept that an unknown part of the population (depending on the actual value of *φ*) evades sampling. However, since we expect *φ* to decline with plant age and size, the bias committed by neglecting the mites on the stems will likely be fairly small when hibiscus plants become older and larger. A protocol based on presence-absence sampling may be a good alternative to counting methods when the purpose is to survey mite populations of *B. yothersi* inhabiting large hibiscus plants. Studies are needed to validate these methods under field conditions so that the method could be used to estimate *B. yothersi* population density for both research and integrated pest management (IPM) applications.

## Electronic supplementary material

Below is the link to the electronic supplementary material.

Supplementary file1 (DOCX 20 KB)

Supplementary file2 (DOCX 43 KB)

## Data Availability

Data is provided within the manuscript and the supplementary information files.

## Appendix 1

### Estimation of mites per plant by sampling both leaves and stems (method 1)

The equations used to estimate the mean number of mites per sampling unit (*μ*) are the same for estimating mites on leaves and stems, so in the following a sampling unit refers to either a leaf or a stem unit.

The potential number of sampling units per stratum is denoted *N*_*h*_ of which ${n}_{h}$ are sampled (i.e. ${n}_{h} \le {N}_{h}$). The number of mites in the *i*th unit taken from the *h*th stratum (*h* = 1,2,..,*L*) is denoted ${y}_{hi}$ (*i* = 1, 2,…,${n}_{h}$). The mean number of mites per sampling unit in stratum *h* is estimated from the sample as

11 $$\hat{\mu }_{h} = \frac{{\mathop \sum \nolimits_{i = 1}^{{n_{h} }} y_{hi} }}{{n_{h} }}$$

while the variance of ${y}_{hi}$ is found as

12 $$s_{h}^{2} = \frac{{\mathop \sum \nolimits_{i = 1}^{{n_{h} }} \left( {y_{hi} - \hat{\mu }_{h} } \right)^{2} }}{{n_{h} - 1}}$$

Instead of calculating ${s}_{h}^{2}$ by means of Eq. 12, we may use the relationship ${s}_{h}^{2}=a{\widehat{\mu }}_{h}^{b}$ (Eq. 4), which allows us to estimate ${s}_{h}^{2}$ even if only a single unit is sampled from stratum *h.*

The overall number of mites per sampling unit is estimated as

13 $$\hat{\mu } = \mathop \sum \limits_{h = 1}^{L} W_{h} \hat{\mu }_{h},$$

where $${W}_{h}={N}_{h}/N$$ and $$N={\sum }_{h=1}^{L}{N}_{h}$$.

The sample variance of $\widehat{\mu }$ is found as (Cochran 1977)

14 $$v\left( {\hat{\mu }} \right) = \mathop \sum \limits_{h = 1}^{L} W_{h}^{2} v(\hat{\mu }_{h} ),$$

where ${v(\widehat{\mu }}_{h})$ is the sample variance of ${\widehat{\mu }}_{h}$, given as

15 $$v\left( {\hat{\mu }_{h} } \right) = \frac{{1 - f_{h} }}{{n_{h} }}s_{h}^{2}$$

${f}_{h}$ is the fraction of all units sampled in the *h*th stratum, i.e. ${f}_{h}={n}_{h}/{N}_{h}$. The term $1-{f}_{h}$ is representing the finite population correction (fpc), which will be 0 when ${n}_{h}={N}_{h}$ and close to 1 when ${n}_{h}\ll {N}_{h}$.

To calculate the total number of mites per plant, we introduce a notation that distinguishes between mites sampled on leaves and stems. Hence, let ${m}_{h}$ and ${n}_{h}$ denote the number of leaves and stem units sampled from the *h*th stratum consisting of ${M}_{h}$ leaves and ${N}_{h}$ stem units, and let $m={\sum }_{h=1}^{L}{m}_{h}$, $n={\sum }_{h=1}^{L}{n}_{h}$, $M={\sum }_{h=1}^{L}{M}_{h}$ and $N={\sum }_{h=1}^{L}{N}_{h}$. Furthermore, let $\overline{x }$ and $\overline{y }$ represent the values of $\widehat{\mu }$ for mites found on leaves and stem units, respectively (Eq. 13). The total number of mites inhabiting a plant is estimated as

16 $$\hat{X} + \hat{Y} = M\overline{x} + N\overline{y},$$

where *X* and *Y* denote the total number of mites occupying the plant’s *M* leaves and *N* stem units, respectively.

Since *X* and *Y* are estimated from two independent samples, we obtain the variance of $\widehat{X}+\widehat{Y}$ as

17 $$v\left( {\hat{X} + \hat{Y}} \right) = v\left( {\hat{X}} \right) + v\left( {\hat{Y}} \right) = M^{2} v\left( {\overline{x}} \right) + N^{2} v\left( {\overline{y}} \right),$$

where $v\left(\overline{x }\right)$ and $v\left(\overline{y }\right)$ are calculated by means of Eq. 14.

The Mean Squared Error (*MSE*) of $\widehat{X}+\widehat{Y}$ is found from Eq. 8 as

18 $$MSE\left( {\hat{X} + \hat{Y}} \right) = v\left( {\hat{X} + \hat{Y}} \right) + B$$

but since the sampling method is unbiased, we may set *B* = 0.

### Estimation of mites per plant by sampling only leaves (Method 2)

Instead of sampling mites on the stems, we suggest to use Eq. 1 as a short-cut for predicting the number of mites on the stems in stratum *h* (*Y*_*h*_) from the estimated number of mites occupying the leaves in the same stratum (${\widehat{X}}_{h}={M}_{h}{\overline{x} }_{h}$). Furthermore, since *β* in Eq. 1 was found to be close to unity, Eq. 19 may serve the purpose of linking ${\widehat{Y}}_{h}$ to ${\widehat{X}}_{h}$ (cf. Eq. 1a)

19 $$\hat{Y}_{h} = \hat{\varphi }\left( {\hat{X}_{h} + 1} \right) = \hat{\varphi }\hat{X}_{h} + \hat{Y}_{0},$$

where $\widehat{\varphi }={e}^{\widehat{\alpha }}$. ${\widehat{Y}}_{0}(=\widehat{\varphi }-1)$ is the expected number of mites on the stems if ${\widehat{X}}_{h}=0$.

The total number of mites occupying the stems of a sampled plant is estimated as

20 $$\hat{Y} = \mathop \sum \limits_{h = 1}^{L} \hat{Y}_{h} = \mathop \sum \limits_{h = 1}^{L} \left( {\hat{\varphi }\hat{X}_{h} + \hat{Y}_{0} } \right)$$

The sample variance of ${\widehat{Y}}_{h}$ depends on the variance associated with both ${\widehat{X}}_{h}$ and $\widehat{\varphi }$, and is found as

21 $$v\left( {\hat{Y}_{h} } \right) = v(\hat{\varphi }(M_{h} \overline{x}_{h} + 1) - 1) = v(\hat{\varphi }(M_{h} \overline{x}_{h} + 1))$$

According to Colquhoun (1971), the product of the two stochastic variables ($\widehat{\varphi }$ and ${M}_{h}{\overline{x} }_{h}$+1) can be obtained as

22 $$\begin{aligned} v\left( {\hat{\varphi }\left( {M_{h} \overline{x}_{h} + 1} \right)} \right) = \left( {M_{h} \overline{x}_{h} + 1} \right)^{2} v\left( {\hat{\varphi }} \right) + \hat{\varphi }^{2} v\left( {M_{h} \overline{x}_{h} + 1} \right) + v\left( {\hat{\varphi }} \right)v\left( {M_{h} \overline{x}_{h} + 1} \right) \\ & & & = \left( {M_{h} \overline{x}_{h} + 1} \right)^{2} v\left( {\hat{\varphi }} \right) + \hat{\varphi }^{2} v\left( {M_{h} \overline{x}_{h} } \right) + v\left( {\hat{\varphi }} \right)v\left( {M_{h} \overline{x}_{h} } \right) \\ & = \left( {M_{h} \overline{x}_{h} + 1} \right)^{2} v\left( {\hat{\varphi }} \right) + \hat{\varphi }^{2} M_{h}^{2} v\left( {\overline{x}_{h} } \right) + M_{h}^{2} v\left( {\hat{\varphi }} \right)v\left( {\overline{x}_{h} } \right) \\ \end{aligned}$$

where $v\left({\overline{x} }_{h}\right)$ can be found from Eq. 15. Since $\widehat{\varphi }={e}^{\widehat{\alpha }}$, we get $\widehat{\alpha }=\text{ln}\widehat{\varphi }$, where $\widehat{\alpha }$ is the estimated intercept of the straight line used to predict ${Y}_{h}$ from ${\widehat{X}}_{h}$ (Fig. 3). The variance of $\widehat{\varphi }$ is found from the relationship (Colquhoun 1971)

23 $$v\left( {\hat{\varphi }} \right) \approx \hat{\varphi }^{2} v(\ln \hat{\varphi }) = \hat{\varphi }^{2} v\left( {\hat{\alpha }} \right)$$

where $v(\widehat{\alpha })$ is the variance of the line’s intercept with the Y-axis. Hence, $v({\widehat{Y}}_{h})$ is estimated as

24 $$v\left( {\hat{Y}_{h} } \right) = \hat{\varphi }^{2} \left[ {\left( {M_{h} \overline{x}_{h} + 1} \right)^{2} v\left( {\hat{\alpha }} \right) + M_{h}^{2} v\left( {\overline{x}_{h} } \right) + M_{h}^{2} v\left( {\hat{\alpha }} \right)v\left( {\overline{x}_{h} } \right)} \right]$$

Finally, the sample variance of the number of mites occupying the stems of a plant is obtained as

25 $$v\left( {\hat{Y}} \right) = \hat{\varphi }^{2} \mathop \sum \limits_{h = 1}^{L} \left[ {\left( {M_{h} \overline{x}_{h} + 1} \right)^{2} v\left( {\hat{\alpha }} \right) + M_{h}^{2} v\left( {\overline{x}_{h} } \right) + M_{h}^{2} v\left( {\hat{\alpha }} \right)v\left( {\overline{x}_{h} } \right)} \right]$$

If $v\left(\widehat{\alpha }\right)$ is 0, Eq. 25 reduces to

26 $$v\left( {\hat{Y}} \right) = \hat{\varphi }^{2} \mathop \sum \limits_{h = 1}^{L} M_{h}^{2} v\left( {\overline{x}_{h} } \right) = \hat{\varphi }^{2} M^{2} \mathop \sum \limits_{h = 1}^{L} W_{h}^{2} v\left( {\overline{x}_{h} } \right)$$

indicating that the standard error of $\widehat{Y}$ (i.e. $\sqrt{v\left(\widehat{Y}\right)}$) increases proportionally with $\widehat{\varphi }$, *M* and $v\left({\overline{x} }_{h}\right)$.

The sample variance of $\widehat{X}+\widehat{Y}$ is found as

27 $$v\left( {\hat{X} + \hat{Y}} \right) = v\left( {\hat{X}} \right) + v\left( {\hat{Y}} \right) = \mathop \sum \limits_{h = 1}^{L} M_{h}^{2} v\left( {\overline{x}_{h} } \right) + \hat{\varphi }^{2} \mathop \sum \limits_{h = 1}^{L} \left[ {\left( {M_{h} \overline{x}_{h} + 1} \right)^{2} v\left( {\hat{\alpha }} \right) + M_{h}^{2} v\left( {\overline{x}_{h} } \right) + M_{h}^{2} v\left( {\hat{\alpha }} \right)v\left( {\overline{x}_{h} } \right)} \right]$$

Estimates of mites per plant based on Method 2 are likely to be inaccurate due to bias, but their relative precision (*RV*) can be found as

28 $$RV = \frac{{\sqrt {v\left( {\hat{X} + \hat{Y}} \right)} }}{{\hat{X} + \hat{Y}}}$$
