# Supplementary material for: Density and distribution of the flat mite (Brevipalpus yothersi) (Acari: Tenuipalpidae) on four Hibiscus varieties: do leaves tell the full story?
Source: Exp Appl Acarol. 2024 Dec 12;94(1):9. doi: 10.1007/s10493-024-00970-z (PMC11638289; doi:10.1007/s10493-024-00970-z)
Supplement: Supplementary file 1 — Supplementary file1 (DOCX 20 KB) [file 10493_2024_970_MOESM1_ESM.docx]

**Supplementary material S1:**

**Table S1A** Average numbers of Brevipalpus yothersi eggs, immature mites, and adults on leaves, stems, and entire plants. Each value is the average (with standard error in parentheses) of mites on eight plants per variety

| **Property** | **Hibiscus variety** | | | |
| --- | --- | --- | --- | --- |
|  | **Seminole Pink** | **President Red** | **Snow Queen** | **Sunny Yellow** |
| Eggs leaves/plant | 13.00 (8.54) | 127.50 (54.88) | 47.88 (23.17) | 144.75 (143.75) |
| Immature mites leaves/plant | 11.75 (7.98) | 93.13 (54.88) | 106.63 (18.53) | 79.00 (106.20) |
| Adults leaves/plant | 9.50 (4.11) | 107.63 (45.40) | 55.50 (20.16) | 88.50 (87.36) |
| Total mites leaves/plant | 34.25 (18.09) | 328.25 (139.71) | 154.88 (60.65) | 339.88 (337.31) |
| Eggs stems/plant | 207.13 (93.87) | 450.25 (165.22) | 146.00 (45.14) | 161.63 (134.49) |
| Immature mites stems/plant | 79.00 (39.95) | 126.5 (34.22) | 177.63 (60.46) | 58.00 (40.21) |
| Adults stems/plant | 25.13 (10.54) | 62.00 (20.25) | 63.63 (17.03) | 28.75 (20.54) |
| Total mites stems/plant | 311.25 (135.90) | 638.75 (214.70) | 389.25 (120.91) | 248.38 (194.63) |
| Eggs/plant | 220.13 (100.83) | 577.25 (207.85) | 193.88 (61.84) | 306.38 (277.26) |
| Immature mites/plant | 90.75 (47.48) | 219.63 (69.50) | 229.13 (73.22) | 164.63 (144.59) |
| Adults/plant | 34.63 (13.84) | 169.63 (61.39) | 121.13 (34.62) | 117.25 (107.39) |
| Total mites/plant | 345.5 (151.40) | 967.00 (333.71) | 544.13 (164.29) | 588.25 (529.06) |

**Table S1B** Average densities (numbers per cm^2^ surface area) of B. yothersi eggs, immature mites, and adults on leaves, stems, and entire plants. Each value is the average (with standard error in parentheses) of mites on eight plants per variety

| **Property** | **Hibiscus variety** | | | |
| --- | --- | --- | --- | --- |
|  | **Seminole Pink** | **President Red** | **Snow Queen** | **Sunny Yellow** |
| Eggs/leaves (cm^-2^) | 0.0093 (0.0062) | 0.0679 (0.0276) | 0.0241 (0.0112) | 0.1045 (0.1040) |
| Immature mites/leaves (cm^-2^) | 0.0077 (0.0047) | 0.0479 (0.0200) | 0.0263 (0.0096) | 0.0771 (0.0768) |
| Adults leaves (cm^-2^) | 0.0070 (0.0027) | 0.0590 (0.0258) | 0.0271 (0.0096) | 0.0639 (0.0632) |
| Total mites/leaves (cm^-2^) | 0.0240 (0.0118) | 0.1748 (0.0719) | 0.0775 (0.0298) | 0.2455 (0.2440) |
| Eggs/stems (cm^-2^) | 1.2270 (0.5569) | 1.5592 (0.5504) | 0.4269 (0.1486) | 0.9554 (0.7958) |
| Immature mites/stems (cm^-2^) | 0.4429 (0.2011) | 0.4511 (0.1156) | 0.5188 (0.1989) | 0.3400 (0.2390) |
| Adults stems (cm^-2^) | 0.1525 (0.0644) | 0.2208 (0.0716) | 0.1898 (0.0568) | 0.1675 (0.1219) |
| Total mites/stems (cm^-2^) | 1.8222 (0.7840) | 2.2311 (0.7163) | 1.1356 (0.3991) | 1.4629 (1.1531) |
| Eggs/plants (cm^-2^) | 0.1531 (0.0701) | 0.2875 (0.1032) | 0.0873 (0.0322) | 0.1952 (0.1789) |
| Immature mites/plants (cm^-2^) | 0.0583 (0.0268) | 0.1084 (0.0303) | 0.1056 (0.0417) | 0.1046 (0.0933) |
| Adults/plants (cm^-2^) | 0.0241 (0.0094) | 0.0829 (0.0302) | 0.0521 (0.0161) | 0.0748 (0.0692) |
| Total mites/plants (cm^-2^) | 0.2355 (0.1005) | 0.4788 (0.1514) | 0.2449 (0.0879) | 0.3746 (0.3413) |
