# Supplementary material for: Density and distribution of the flat mite (Brevipalpus yothersi) (Acari: Tenuipalpidae) on four Hibiscus varieties: do leaves tell the full story?
Source: Exp Appl Acarol. 2024 Dec 12;94(1):9. doi: 10.1007/s10493-024-00970-z (PMC11638289; doi:10.1007/s10493-024-00970-z)
Supplement: Supplementary file 2 — Supplementary file2 (DOCX 43 KB) [file 10493_2024_970_MOESM2_ESM.docx]

**Supplementary material S2: Description of Excel program used for simulating computing sampling of *Brevipalpus yothersi* occupying leaves and stems of four hibiscus cultivars.**

The file “Sampling of mites.xlsx” contains counts of mites occurring on all leaves and stem units obtained from each stratum of 32 small hibiscus plants. Sampling of mites can be simulated by assuming that $m_{h}$ leaves and $m_{h}$ stem units are collected at random from stratum *h* (*h =* 1,2,3) in each of the 32 plants. Strata are numbered from below (bottom, middle and top). For a given allocation of sampling units among the strata, the expected values of *RMSE* (Relative Mean Square Error) and sampling time (*T*), averaged over the 32 plants, are calculated. Two different sampling methods are simulated: Method 1 requires that at least one leaf and one stem are sampled per stratum, while Method 2 requires that at least one leaf per stratum is sampled. The number of leaves and stems sampled per plant are found as $m=\sum_{h=1}^{L} m_{h}$ and $n=\sum_{h=1}^{L} n_{h}$, respectively, where *L* denotes the number of strata (i.e. *L =* 3). The allocation of sampling units among strata can be either equal (i.e. $m_{h}=m/L$; $n_{h}=n/L$) or optimal (the allocation that minimizes *RMSE* for a given number of sampling units per plant).

Method 1:

Cells in the spreadsheet “Method 1” are marked in different colors. By default all colored cells except the yellow ones are protected and cannot be changed unless the spreadsheet becomes unprotected (see below). The yellow cells allow for entering user-defined values. Light yellow cells (B3:B4 and B10:B14) contain the default values of the model’s parameters (Table 3 in the main text). Dark yellow cells contain information about $m_{h}$ (O123:Q123) and $n_{h}$ (AF123:AH123). When these cells change values, the new values of *RMSE* and *T,* averaged over all the 32 plants, are shown in the box comprising O123:BH123. The simulation results for a given allocation are saved by means of copy/paste the values in the box and saving them in a row below. Use the option “Paste Values” to save the numerical values only. The saved values are also shown as curves in the figures to the right.


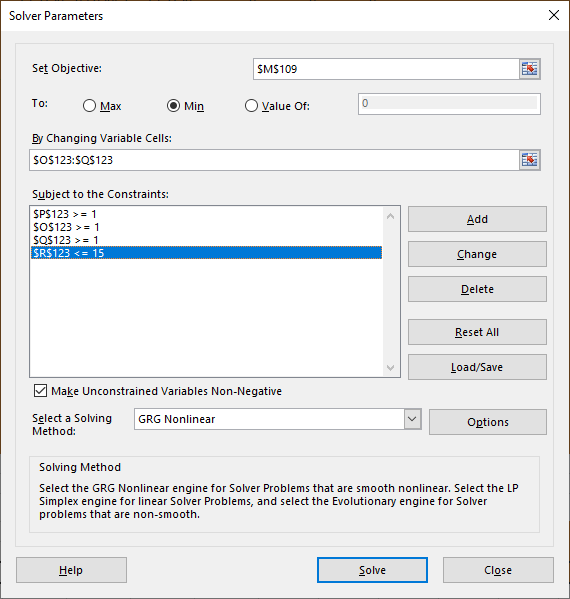
*How to optimize allocation of sampling units*. The optimization process relies on the Solver tool. Unfortunately, Solver does not work on a protected worksheet, so the spreadsheet first has to be unprotected by right-clicking its tab and then choose “Unprotect sheet”. Solver is opened by first clicking on “Data” in the main menu and then choosing “Solver”. The parameters in the Solver window (shown below) depend on whether it is the *m* leaves or the *n* stem units sampled per plant that should be optimized. In the first case, the appropriate parameters are loaded from the cells in BM101:BM108, and in the latter case from cells in BO101:BO108. The figure shows the parameters for optimizing the allocation of 15 leaves (i.e. *m =* 15) sampled per plant. For any given value of *m*, the constraint imposed on cell $R$123 has to be changed so it reads $R$123 ≤ *m.* Likewise, if the model for optimizing stem units is used, the corresponding constraint is set to $AI$123 ≤ *n.* The predicted optimal allocation usually consists of non-integer values, while an actual allocation requires integer values. To achieve this, the values have to be rounded to the nearest integers, but under the constraint that their sum should equal *m* or *n.*

Method 2:

The spreadsheet called “Method 2” is similar to the one used to Method 1, except that only leaves are supposed to be sampled. Instead, the expected number of mites occupying the stems is estimated from the number of mites found on the sampled leaves. It means that only the numbers of sampled leaves per stratum ($m_{1}$, $m_{2}$ and $m_{3}$) need to be entered in the yellow cells O123:Q123.

*How to optimize allocation of sampling units*. As before, the spreadsheet has to be unprotected. The default Solver parameters are loaded from cells AW101:AW108. To find the optimal allocation of *m* leaves sampled per plant, set $R$123 ≤ *m.*
